# Gastric Perforation Secondary to a Hyperinflated Intragastric Balloon: A Case Report and Management Approach

**DOI:** 10.7759/cureus.79928

**Published:** 2025-03-02

**Authors:** Solomon Raj Vasudayan, Guo Hou Loo, Guhan Muthkumaran, Nik Ritza Kosai

**Affiliations:** 1 Upper Gastrointestinal and Metabolic Surgery Unit, Department of Surgery, Faculty of Medicine, The National University of Malaysia, Kuala Lumpur, MYS

**Keywords:** endobariatrics, minimally invasive surgery, obesity, primary repair of gastric ulcer, weight management

## Abstract

Intragastric balloon (IGB) placement is a widely used, minimally invasive intervention for obesity and metabolic disorders, offering a temporary, reversible alternative for weight management. It is generally well tolerated, with most complications being mild and self-limiting, such as nausea, vomiting, and abdominal discomfort. However, in rare cases, more serious complications can arise, including gastric ulceration, balloon migration, and, in extreme cases, gastric perforation. The latter can occur at any time from days to months after insertion, necessitating prompt recognition and surgical intervention to prevent life-threatening consequences. We report a case of a 47-year-old woman who presented with an acute abdomen four months after IGB insertion. Imaging revealed a hyperinflated IGB with associated gastric perforation. The patient was urgently taken to the operating room, where an on-table esophagogastroduodenoscopy confirmed the findings. Laparoscopic primary repair was performed following balloon removal. The mechanism behind IGB hyperinflation remains multifactorial. Computed tomography is the preferred imaging modality for diagnosis. In bariatric centers, a minimally invasive approach, combining endoscopic balloon removal with laparoscopic perforation repair, has demonstrated superior outcomes compared to open surgery, reducing morbidity and recovery time. Endoscopic balloon removal combined with laparoscopic repair offers significant advantages, including minimal scarring, faster recovery, and shorter hospital stays. Early detection and a multidisciplinary approach are crucial for optimal patient outcomes.

## Introduction

Obesity and metabolic disorders are global health challenges, and intragastric balloon (IGB) placement has emerged as a minimally invasive, reversible intervention for weight management [1]. The procedure involves the endoscopic insertion of a balloon into the stomach, which is then inflated with saline and methylene blue to restrict gastric capacity and promote early satiety, thereby inducing weight loss [2,3]. Various IGB types are available, including traditional nonadjustable models and adjustable variants such as the Spatz3 balloon [4]. The Spatz3 balloon is typically filled with 500 mL of methylene blue-stained sterile water under aseptic conditions and can remain in situ for up to one year [4].

Proper maintenance of the IGB is crucial in preventing complications. Patients are advised to adhere to dietary modifications, avoid excessive gastric irritants, and take prescribed proton pump inhibitors (PPIs) to reduce gastric acid secretion and minimize mucosal injury [5]. PPIs play a key role in preventing gastric ulceration, which is a recognized risk factor for balloon-related perforation [5]. Additionally, close monitoring for symptoms of intolerance or potential complications, such as persistent epigastric pain, nausea, or reflux, is essential for early detection and timely management.

Although IGB placement is generally safe, complications can arise, with gastric perforation being a rare but potentially life-threatening event. Perforation is often attributed to prolonged mechanical pressure on the gastric mucosa, leading to ulcer formation and eventual transmural injury [1]. Additionally, IGB hyperinflation, a phenomenon reported in the United States, has been linked to gas-producing microorganisms, though its exact pathophysiology remains unclear [3]. To date, no cases of IGB hyperinflation have been documented in Malaysia.

This case report describes a patient who developed a perforated gastric ulcer secondary to hyperinflation of an IGB. The condition was successfully managed using a combined endoscopic and laparoscopic approach, highlighting the importance of early recognition and minimally invasive intervention in optimizing patient outcomes.

## Case presentation

A 47-year-old woman presented with a sudden-onset, severe, generalized, non-radiating abdominal pain. She had undergone the placement of an adjustable IGB (Spatz3) four months earlier, with an initial inflation volume of 500 mL of sterile water. Her body mass index (BMI) had decreased from 35.9 kg/m² at the time of insertion to 29.8 kg/m² over four months.

**Figure 1:**
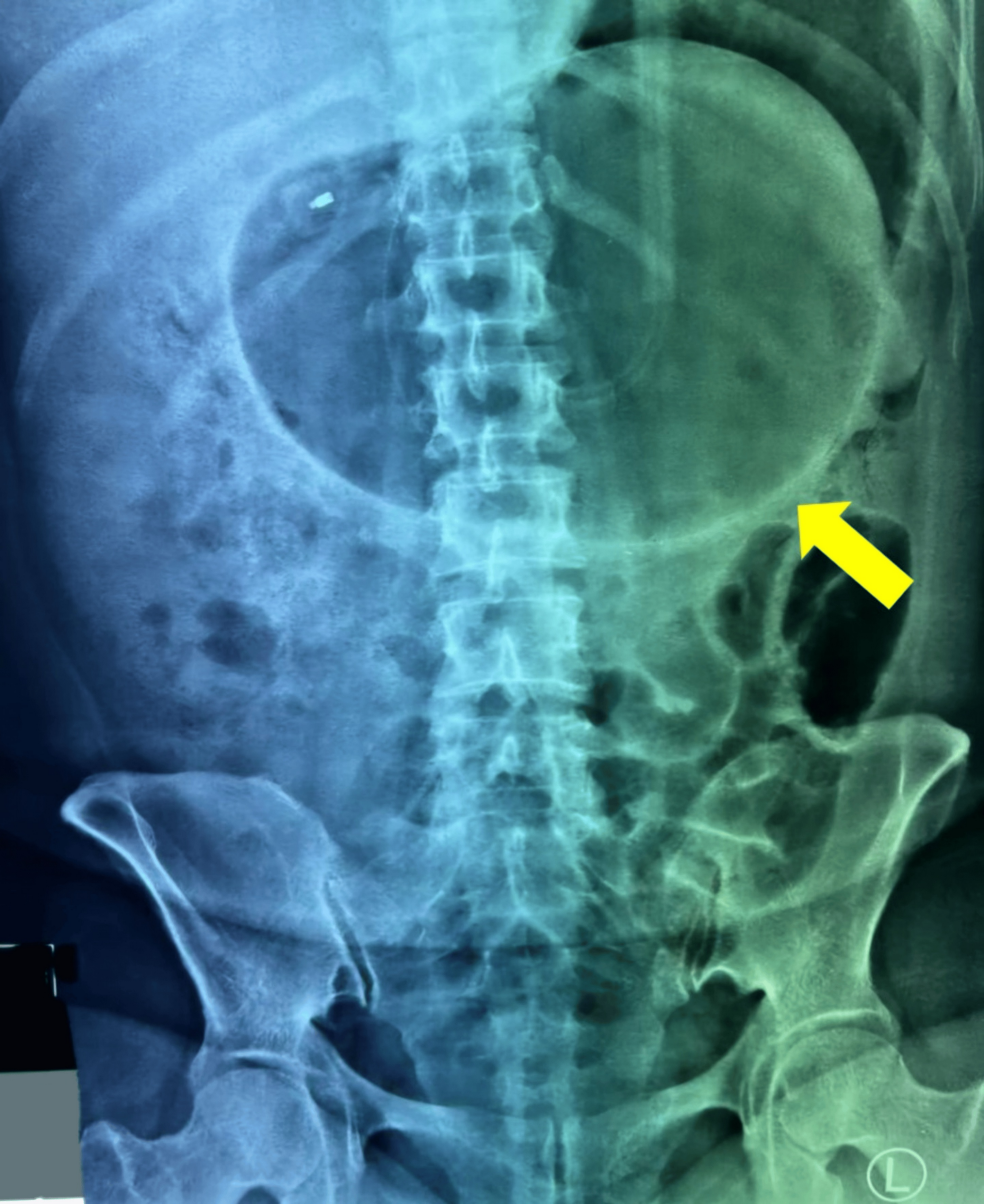
Plain abdominal X-ray showing a hyperinflated intragastric balloon (yellow arrow) occupying almost half of the abdominal cavity.

Upon arrival at the emergency department, she was afebrile but dehydrated, tachycardic, and normotensive. An abdominal examination revealed epigastric fullness, tenderness, and localized guarding. A plain abdominal X-ray demonstrated a grossly overdistended IGB occupying almost half of the abdominal cavity (Figure 1). However, the X-ray did not include the domes of the diaphragm, which limited its ability to detect pneumoperitoneum. A chest X-ray in an erect position was not performed, as the decision was made to proceed with a contrast-enhanced computed tomography (CECT) scan for a more thorough assessment following the identification of the hyperinflated balloon.

**Figure 2:**
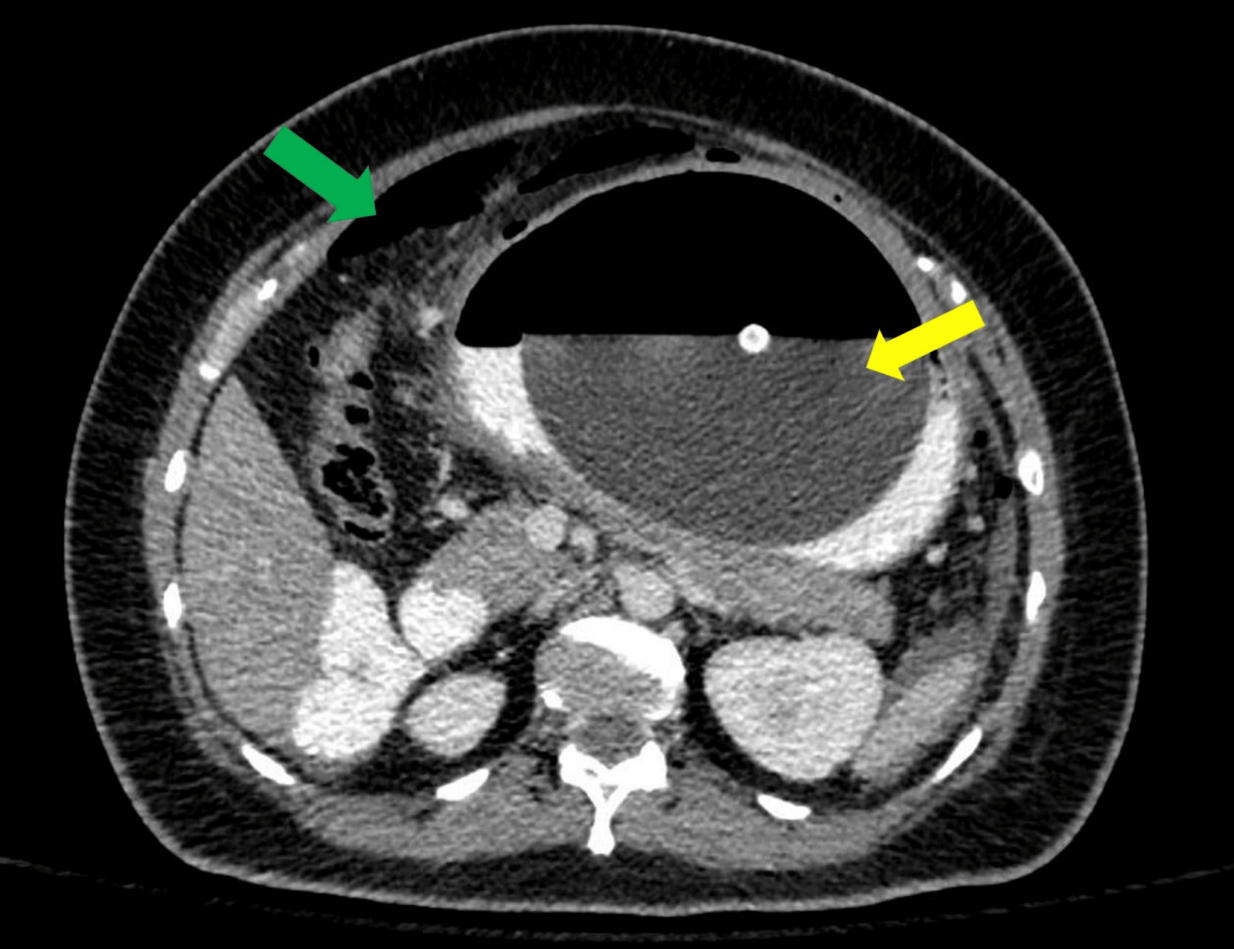
Contrast-enhanced CT of the abdomen (axial view) showing the hyperinflated intragastric balloon within the stomach (yellow arrow) with surrounding oral contrast and pneumoperitoneum (green arrow).

**Figure 3:**
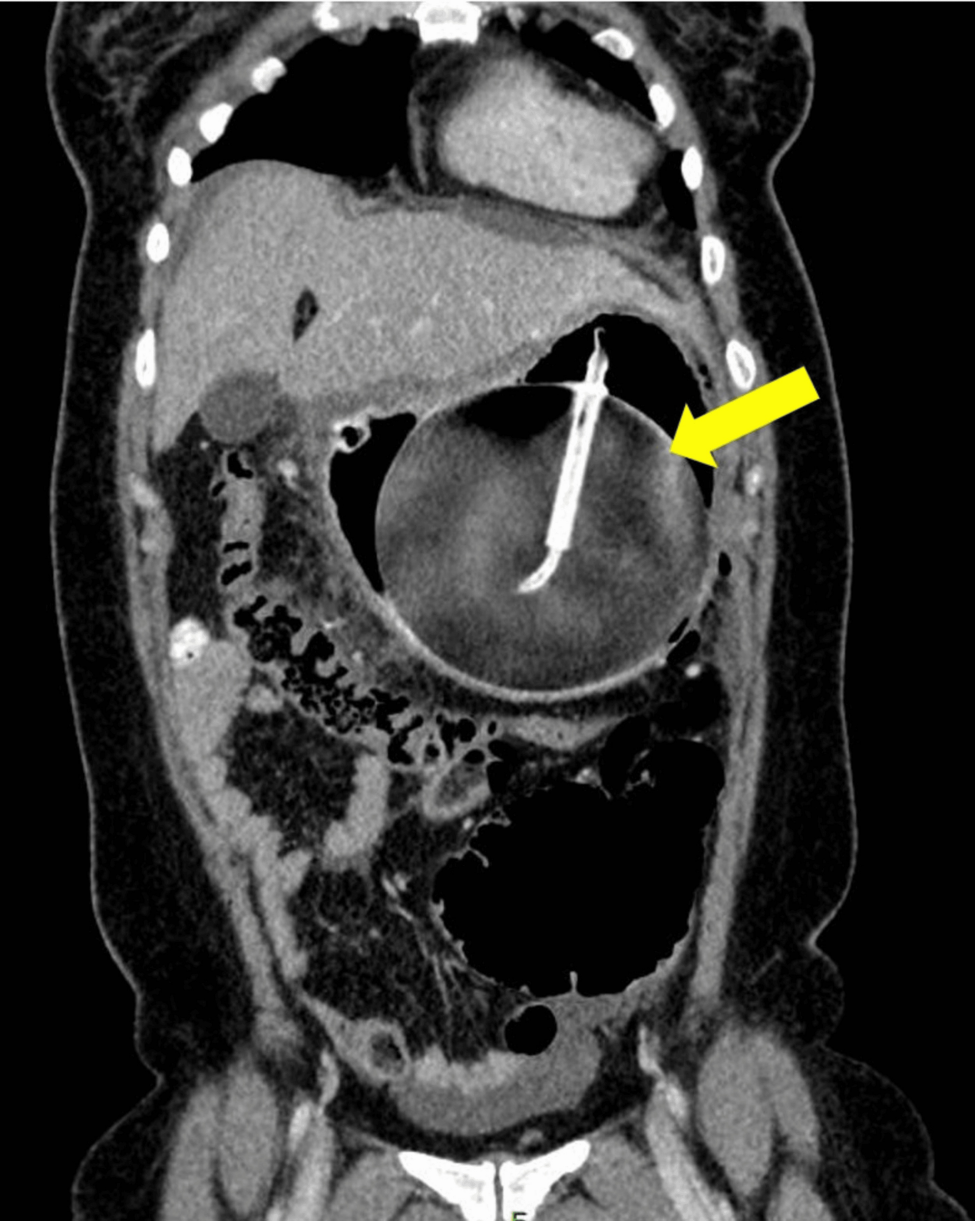
Contrast-enhanced CT of the abdomen (coronal view) showing the hyperinflated intragastric balloon (yellow arrow) within the stomach occupying the entire upper quadrant of the abdomen.

The CT scan confirmed the presence of pneumoperitoneum, gastric wall thickening with air pockets, fat stranding, and free intraperitoneal fluid, suggestive of perforated viscus (Figures 2, 3). While an erect chest X-ray may have helped detect free air under the diaphragm, CT imaging was prioritized due to its superior sensitivity in evaluating the extent of the perforation and associated intra-abdominal pathology, which was crucial for surgical planning.

**Figure 4:**
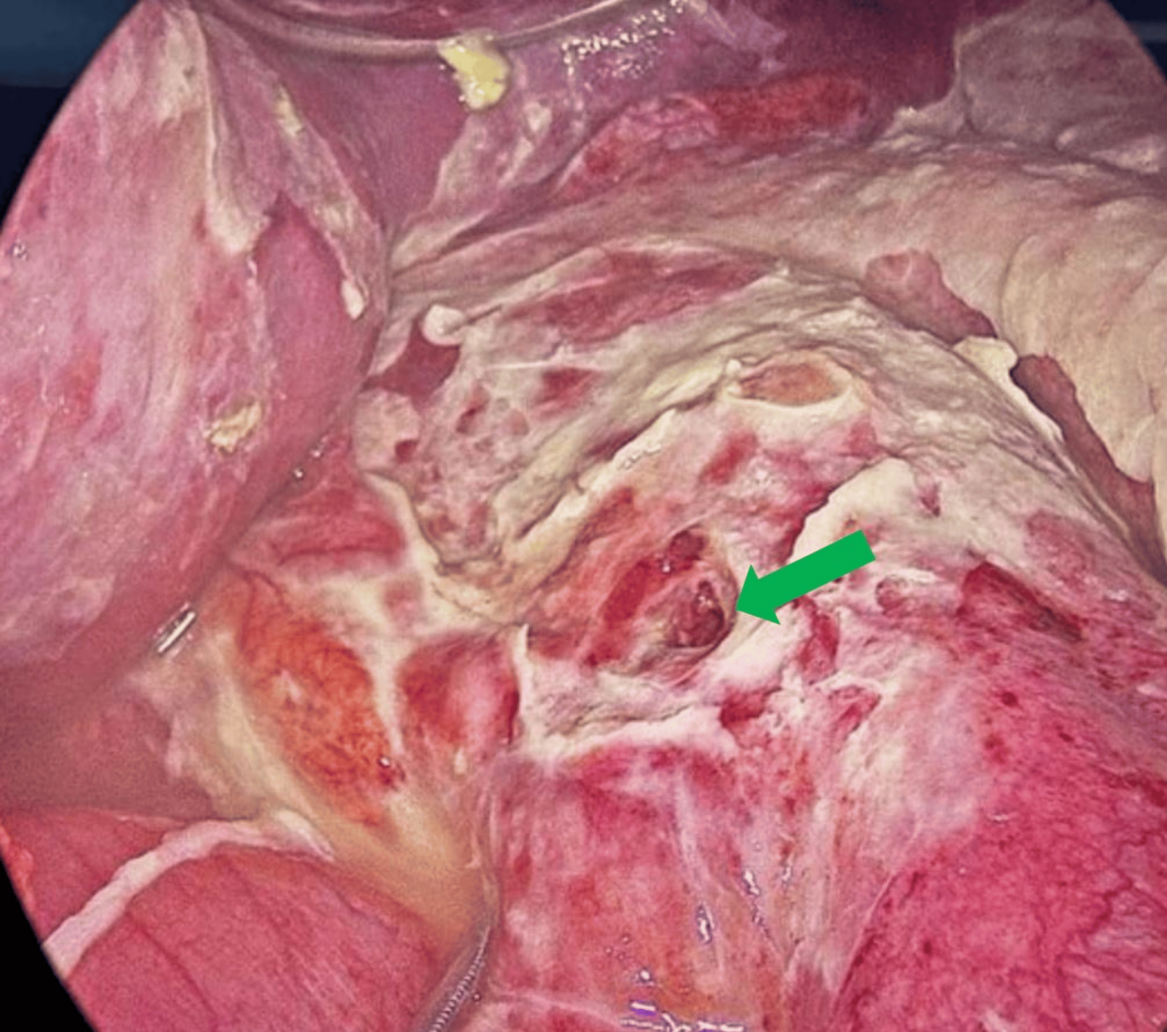
Intraoperative image showing a 1 cm prepyloric perforation (green arrow) over the anterior wall of the stomach with surrounding contamination.

The patient was promptly taken to the operating theater, where on-table esophagogastroduodenoscopy (OGDS) confirmed the presence of a hyperinflated IGB. The balloon was deflated via aspiration of 500 mL of fluid along with gas bubbles and was removed endoscopically. Laparoscopic exploration revealed a 1 cm pre-pyloric gastric perforation with gross purulent contamination extending to all quadrants of the abdomen (Figure 4).

A laparoscopic primary repair of the gastric ulcer was performed using Stratafix™ Symmetric PDS™ Plus Knotless Tissue Control Device (Ethicon, Raritan, NJ) and reinforced with a pedicled greater omentum.

The aspirated IGB fluid was sent for culture and sensitivity, but no bacterial or fungal growth was identified. Postoperatively, the patient was closely monitored and gradually reintroduced to oral feeding. She showed an uneventful recovery and underwent *Helicobacter pylori* eradication therapy. A repeat OGDS performed a month later showed a well-healed gastric ulcer with no evidence of malignancy.

## Discussion

The most commonly reported complications of IGB are abdominal pain (33.7%), nausea (29%), and gastroesophageal reflux (18.3%). Besides these, there were reported balloon migration and small bowel obstruction (0.8%) cases. Gastric ulcers developed in about 0.4% of patients, and perforation developed in about 0.1% of patients [1]. Gastric ulcer and perforation are the most serious complications of IGB.

Hyperinflated IGB may present with mild epigastric discomfort, vomiting, and bloating [3]. Stomach perforation post IGB may present with mild to severe epigastric tenderness with localized guarding to generalized peritonitis, associated with vomiting. A common reason for perforation is non-compliance to PPIs post insertion [1,6]. Our patient is the first reported case of stomach perforation due to hyperinflated IGB whilst compliant with her PPI with no other risk factors associated with stomach perforation or gastric ulcers.

The exact mechanism underlying spontaneous IGB hyperinflation remains unclear. A systematic review and ex vivo study by Hawa et al. explored possible causes, hypothesizing both iatrogenic and mechanical factors. These include inadvertent overinflation during insertion and structural defects such as a malfunctioning balloon valve that may allow the ingress of gas over time [7]. Additionally, the fermentation of intraluminal contents within the balloon has been postulated as a potential mechanism contributing to hyperinflation, although this remains controversial and lacks definitive evidence [3]. Some cases were reported to have contamination of the IGB fluid with gas-forming microorganisms like some species of *Candida* and *Streptococcus viridans*, leading to hyperinflation. Hyperinflation of IGB happens when the balloon is filled with air component while the fluid level remains the same. In our patient, the fluid was filled in a sterile manner, and no bacteria and/or fungi were isolated in the balloon fluid culture and sensitivity postoperatively. The sensitivity of fluid culture and sensitivity in detecting spore-forming bacteria/fungi remains a question, as not every hyperinflated IGB case has reported isolation of organisms.

An additional factor that may contribute to the relative hyperinflation of the balloon is weight loss-induced gastric volume reduction. As BMI decreases, the stomach size is expected to shrink, potentially leading to increased balloon-to-stomach ratio and excessive mucosal pressure. This theory is supported by previous observations that smaller gastric cavities may be more prone to complications such as gastric ulcers and perforation due to increased mechanical strain from the balloon [8]. In our patient, the degree of weight loss and gastric volume reduction may have played a role in increasing intragastric pressure, thereby predisposing to perforation. Future studies evaluating the relationship between weight loss, gastric capacity changes, and IGB complications may help refine current balloon volume recommendations to minimize such risks.

The most common side of gastric perforation following a complication of IGB is at the lower anterior gastric body. This is because the lower body and the angle of the stomach are more prone to pressure ulceration due to the balloon’s continuous contact with the gastric wall, leading to a decrease in the microcirculation in these areas [1]. In our patient, the perforation was at the pre-pyloric region, anterior surface. It is recommended that balloons should not stay in place for more than one year for Spatz3 balloon, and patients should use PPIs post procedure. Prior to the procedure, OGDS and *H. pylori* testing are recommended, with the initiation of treatment if positive, for the prevention of peptic ulcer and stomach perforation [5]. Any prior gastric surgery resulting in an anatomical abnormality of the gastrointestinal tract is an absolute contraindication for the use of IGB as the compliance of the stomach may be modified [5,9].

In cases of hyperinflated IGB, an abdominal X-ray can help outline hyperinflation. In cases of early perforation, we may not see air under the diaphragm in an erect chest X-ray. Hence, a CT scan would be the better choice of imaging modality to identify pneumoperitoneum early, thereby preventing delay in diagnosing, worsening symptoms, and peritonism, which may require laparotomy. With early diagnosis, minimally invasive surgeries, endoscopic closure, and endoclips have shown success in managing stomach perforation [2]. Early detection of hyperinflation is critical in preventing complications such as gastric perforation. Symptoms that may suggest balloon hyperinflation include progressive epigastric discomfort, worsening bloating, nausea, early satiety, and reflux-like symptoms. Some patients may also experience increased belching or a sensation of excessive gastric distension. On physical examination, findings such as increased epigastric tenderness or a palpable upper abdominal mass could raise suspicion of balloon overexpansion [5].

Radiological and endoscopic evaluations can aid in the early detection of hyperinflation. A simple abdominal X-ray may reveal an abnormally large gas-filled balloon, while ultrasound or fluoroscopy with contrast may help assess balloon size and positioning. Endoscopic evaluation can be particularly useful if patients develop persistent symptoms, as it allows direct visualization of mucosal changes that might suggest excessive pressure from the balloon [9]. Preventative measures include patient education on recognizing early symptoms, scheduled clinical follow-ups, and possible periodic imaging to assess balloon status in high-risk cases. Additionally, refining balloon volume recommendations based on individual patient factors such as BMI reduction and gastric capacity changes could help minimize the risk of hyperinflation-related complications [9].

In case of perforation, many authors described a laparotomy for the device removal and the repair of the defect [6,9]. The rate of mortality after surgery (laparoscopic and laparotomy) accounts for 16.6%, whereby all reported cases were done via laparotomy. There is one case that was reported to have successful conservative management. However, the high mortality rate and complication risk raise doubts regarding conservative management [9]. There were cases reported using minimally invasive techniques, a combination of endoscopic-laparoscopic procedure in bariatric centers, which allows balloon removal and visualization of the perforation by pinpointing its precise location [9]. In our patient, a combined procedure of on-table OGDS and laparoscopic modified Graham patch repair was successful in removing the balloon and managing the perforation. This allowed her to leave the hospital in a stable condition after establishing oral feeding and ensuring a rapid return to social life and work.

## Conclusions

Gastric perforation is a rare but life-threatening complication of IGB placement. Clinicians should maintain a high index of suspicion for perforation in patients presenting with acute abdominal symptoms following IGB insertion. Early diagnosis, facilitated by imaging and endoscopic assessment, is crucial for timely intervention. A minimally invasive approach offers significant advantages. This case highlights the importance of prompt recognition and multidisciplinary management in optimizing patient outcomes.
